# An Oximetry Based Wireless Device for Sleep Apnea Detection

**DOI:** 10.3390/s20030888

**Published:** 2020-02-07

**Authors:** Fábio Mendonça, Sheikh Shanawaz Mostafa, Fernando Morgado-Dias, Antonio G. Ravelo-García

**Affiliations:** 1 Instituto Superior Técnico, University of Lisbon, 1049-001 Lisbon, Portugal; sheikh.mostafa@tecnico.ulisboa.pt; 2 ITI/Larsys/Madeira Interactive Technologies Institute, 9020-105 Funchal, Portugal; antonio.ravelo@ulpgc.es; 3 Faculty of Exact Sciences and Engineering, University of Madeira, 9000-082 Funchal, Portugal; 4 Institute for Technological Development and Innovation in Communications, Universidad de Las Palmas de Gran Canaria, 35001 Las Palmas de Gran Canaria, Spain

**Keywords:** OSA, apnea, SpO2, home monitoring device

## Abstract

Sleep related disorders can severely disturb the quality of sleep. Among these disorders, obstructive sleep apnea (OSA) is highly prevalent and commonly undiagnosed. Polysomnography is considered to be the gold standard exam for OSA diagnosis. Even though this multi-parametric test provides highly accurate results, it is time consuming, labor-intensive, and expensive. A non-invasive and easy to self-assemble home monitoring device was developed to address these issues. The device can perform the OSA diagnosis at the patient’s home and a specialized technician is not required to supervise the process. An automatic scoring algorithm was developed to examine the blood oxygen saturation signal for a minute-by-minute OSA assessment. It was performed by analyzing statistical and frequency-based features that were fed to a classifier. Afterward, the ratio of the number of minutes classified as OSA to the time in bed in minutes was compared with a threshold for the global (subject-based) OSA diagnosis. The average accuracy, sensitivity, specificity, and area under the receiver operating characteristic curve for the minute-by-minute assessment were, respectively, 88%, 80%, 91%, and 0.86. The subject-based accuracy was 95%. The performance is in the same range as the best state of the art methods for the models based only on the blood oxygen saturation analysis. Therefore, the developed model has the potential to be employed in clinical analysis.

## 1. Introduction

The quality of sleep examination is getting more relevance in the current healthcare systems since sleep related complaints are the second most common reason for pursuing medical care that is only superseded by the feel of pain [1]. It was also forecasted that non-restorative sleep would impact the world economy due to a decrease in workplace productivity. As an example, a reduction between 299 billion and 433 billion dollars is expected for the United States of America by the year 2020 [2].

Typically, poor sleep quality is directly associated with the occurrence of a sleep related disorder (more than 60 disorders have been identified by the International Classification of Sleep Disorders) [3]. Among these disorders, the sleep related breathing disorders are the most prevalent and obstructive sleep apnea (OSA) is the most common in the adult population. It is characterized by a partial or complete obstruction of the upper airway that disrupts the ventilation during sleep [3]. The severity of the disorder is commonly assessed by the apnea-hypopnea index (AHI) that is given by the ratio of the number of apnea and hypopnea events per hour of sleep [3].

The prevalence of the OSA in the 30 to 49 year-old female and male population was estimated to be, respectively, 3% and 10% considering an AHI ≥ 15 events/hour [4]. These incidences increase with age, affecting respectively 9% and 17% of the 50 to 70 year-old female and male population [4]. Nevertheless, it was estimated that most cases (around 80%) are undiagnosed, often due to the lack of information about the disorder or absence of resources to perform the examination [5]. 

Polysomnography (PSG) is considered the gold standard for sleep analysis. It is a multi-parametric test that records multiple signals, such as the blood oxygen saturation (SpO2) [6,7], which are analyzed by a specialized technician to perform clinical diagnoses [8]. Even though the exam produces highly accurate results, it is labor-intensive [9], expensive [10], and usually has a long waiting period for schedule [11]. The hypothesis, considered in this work, is that these issues associated with the OSA diagnosis, can be addressed by a home monitoring device (HMD) capable of performing the diagnosis using a non-invasive and easy to self-assemble sensor, such as a pulse oximeter that can measure the SpO2 signal. Such devices can be categorized as a Type IV according to the American Academy of Sleep Medicine categorization [12]. The hypothesis is supported by the conclusions of two reviews, one that examined HMD for OSA examination [13] and the other that examined proposed methods for the most common source sensors employed for OSA diagnosis [14]. It was verified that combining source sensors did not contribute to a substantial increase of the classification capability, suggesting that one of the sensors is dominating the examination. It was also concluded that research-based devices are concentrating the analysis on the SpO2 and sound signals. Another relevant conclusion was that electrocardiography (ECG) and SpO2 are possibly the most relevant sensors for OSA analysis. Therefore, the proposed methodology comprises the OSA diagnosis based only on the SpO2 signal analysis.

Therefore, an algorithm for OSA examination, based on the SpO2 signal, was developed. The proposed method first determines the epochs where OSA happened and, afterward, this information was employed to perform the OSA diagnosis through a threshold-based classification. The developed algorithm was implemented in a HMD that can perform the OSA examination in the patient’s home without the need for attendance from a technician (to monitor the subject or help assembly the device). The device can be used to perform the OSA diagnosis, help the scheduling of PSG by prioritizing the positive cases for the exam and help in the follow-up treatment of the disorder.

Hence, the goal of this work was the development and implementation in a HMD of an OSA detection algorithm, using only one sensor that is non-invasive and simple to self-assemble.

The paper is organized as follows: Section 2 presents the materials and methods; Section 3 presents the performance of the developed algorithm; Section 4 presents the discussion of the results; Section 5 presents the developed HMD; Section 6 presents the conclusion and future work.

## 2. Materials and Methods

**Figure 1:**
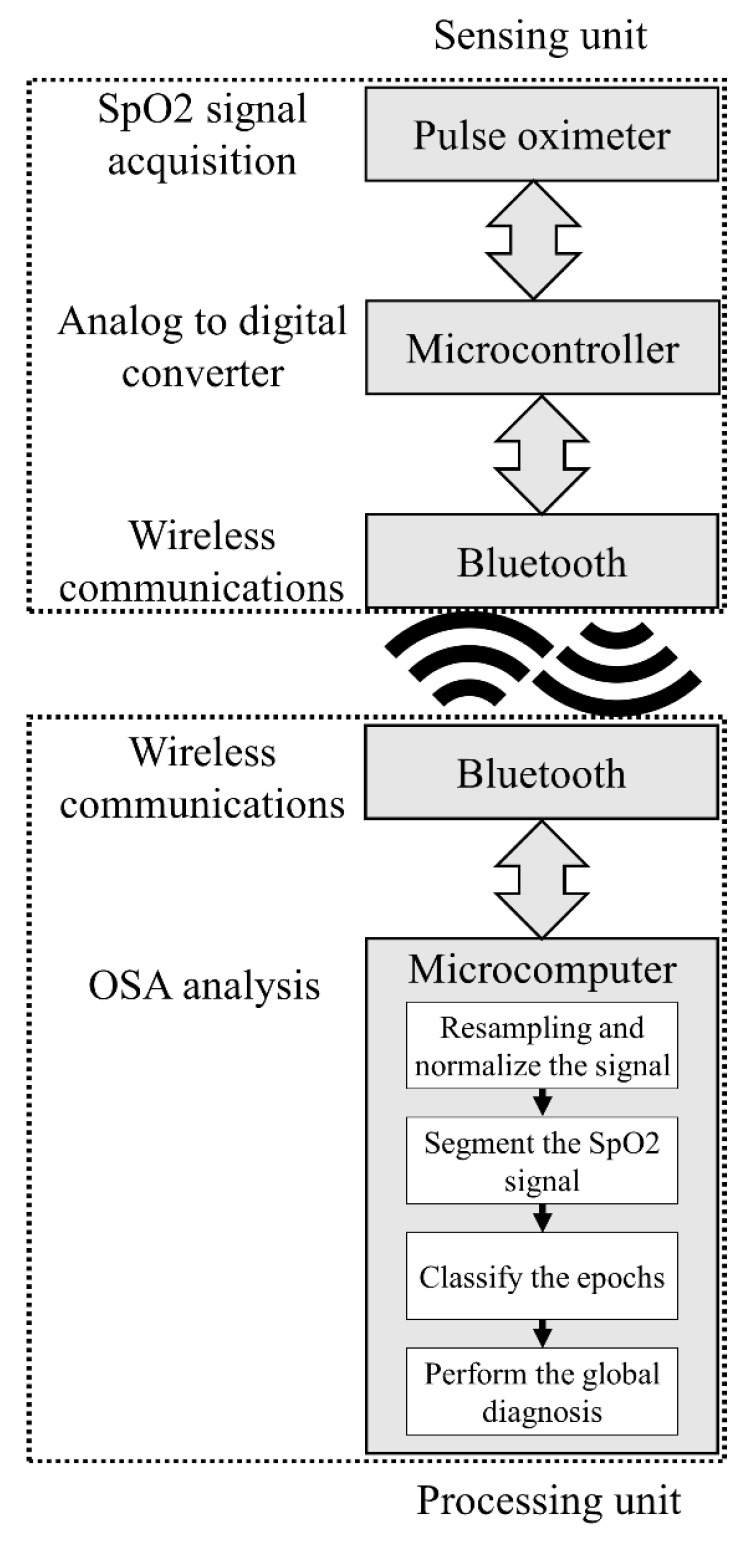
Architecture of the developed HMD.

The SpO2 signal was analyzed by producing statistical and frequency-based features, for each five-minute epoch, which were fed to a logistic regression (LR) for OSA classification. The total number of OSA events were then considered to achieve a global OSA diagnosis by comparing the ratio of the number of minutes classified as OSA to the time in bed in minutes (m-AHI-tib) with a threshold. The algorithm (developed in Python 3) was implemented in the HMD whose architecture is presented in Figure 1. The developed device is composed of a sensing unit, for signal acquisition that is wirelessly sent to a processing unit that performs the OSA examination.

### 2.1. Database

Full night sleep recordings, collected by the sleep unit of the Hospital Universitario de Gran Canaria Dr. Negrín, were employed to develop the OSA detection algorithm [15]. A total of 70 suspected OSA patients were recorded (19 females and 51 males), and the age ranges from 18 and 82 years old. A specialized physician annotated the respiratory events every minute and the recording’s length ranged from 230 to 486 min. An AHI of 5 or more occurred in 50 recordings (diagnosed OSA patients) while the remaining subjects were controls (AHI lower than 5). The SpO2 signals were recorded with the Adult SpO2 sensor (Nellcor, Minneapolis, MN, USA) using a sampling rate of 50 Hz and a resolution of 16 bits [16]. 

### 2.2. Pre-Processing

The sampling rate of the sensor used in the hospital recordings (50 Hz) was lower than the sampling frequency of the sensor employed in the developed HMD (100 Hz). Therefore, the hospital recordings were resampled to 100 Hz by interpolation [17]. Afterward, the signal was normalized, by subtracting the average and dividing the result by the standard deviation, to improve the classification performance [18].

For the minute-by-minute assessment, a 5 min epoch with 1 min displacement between adjacent frames, was employed since it was identified as an appropriate duration for OSA detection [16].

### 2.3. Feature Creation

A total of 22 features were created for each recording. Two of them are from the time domain and examined the variance, defined as

(1) $$V=\frac{1}{N}\overset{N}{\underset{i=1}{\sum }}\left(x_{i}-\bar{x}\right)$$

where *N* is the number of epochs, *x* denotes the samples of the epoch and $\bar{x}$ is the average of the recording. Specifically the variance of the central minute of the five-minute epoch and variance of the five-minute epoch were examined. The other 20 features were from the frequency domain and correspond to the output of each filter from a 20 equally spaced filter bank, defined as [16]

(2) $$Y\prime \left(n\right)=\frac{\sum_{l=b_{n}-\sigma_{n}}^{b_{n}+\sigma_{n}}S\left(l\right)R_{\sigma_{n}}\left(l\right)}{\sum_{l=0}^{\frac{N}{2}-1}S\left(l\right)}$$

where *R* is the rectangular windowing process that was applied in each filter *n* (number of filters ranged from 1 to 20) with bandwidth *σ_n_*. It was considered that *σ_n_* = *σ* ∀ *n* and *b* is the central frequency of the *n*-th band, over the periodogram calculated by [16]

(3) $$S\left(l\right)=\frac{1}{M}{\left|\overset{M-1}{\underset{n=0}{\sum }}s\left(m\right)e^{\frac{-j2\pi lm}{M}}\right|}^{2}$$

where *M* designates the number of samples of *S*(*l*). The analysis covers the whole frequency band of the 5 min epoch and each filter output was normalized to avoid dependencies with the signal dynamic. The bandwidth of each filter was 1.25 Hz.

After the creation of the features, a dynamic compression was applied, using the Neperian logarithm, to make the system more resilient to dynamic changes [16].

### 2.4. Classification

A LR was employed, modeling the probability of the disorder as a function of the selected features by [15]

(4) $$P_{OSA}=\frac{1}{1+e^{-\left(\beta_{0}+\beta_{1}x_{1}+\ldots +\beta_{n}x_{n}\right)}}$$

where *x*_1_…*x_n_* are the selected features and *β*_1_…*β_n_* are the trained model parameters (weights) [19]. The subsequent probability was employed to categorize the epoch as either non-OSA or OSA according to a threshold-based diagnostic rule (the select threshold was also 0.5 since it is a binary classification). Afterward, the global diagnosis of the disorder was performed by examining the ratio of the number of classified OSA epochs in minutes to the time in bed in minutes. This ratio was designated m-AHI-tib since it is highly correlated with the AHI [16,20,21], and it has the advantage that it does not require a sleep/wake classification.

The m-AHI-tib ratio was compared with a threshold to perform the OSA diagnosis. The chosen threshold was 0.083 since it is correlated with an AHI of 5 (minimum value to diagnose OSA, thus 5/60 ≈ 0.083 that represents an AHI of 5 in 60 min). Hence, the epoch-based output of the classifier (minute-by-minute classification) allows the creation of a global score that corresponds to an AHI greater than or equal to 5.

**Figure 2:**
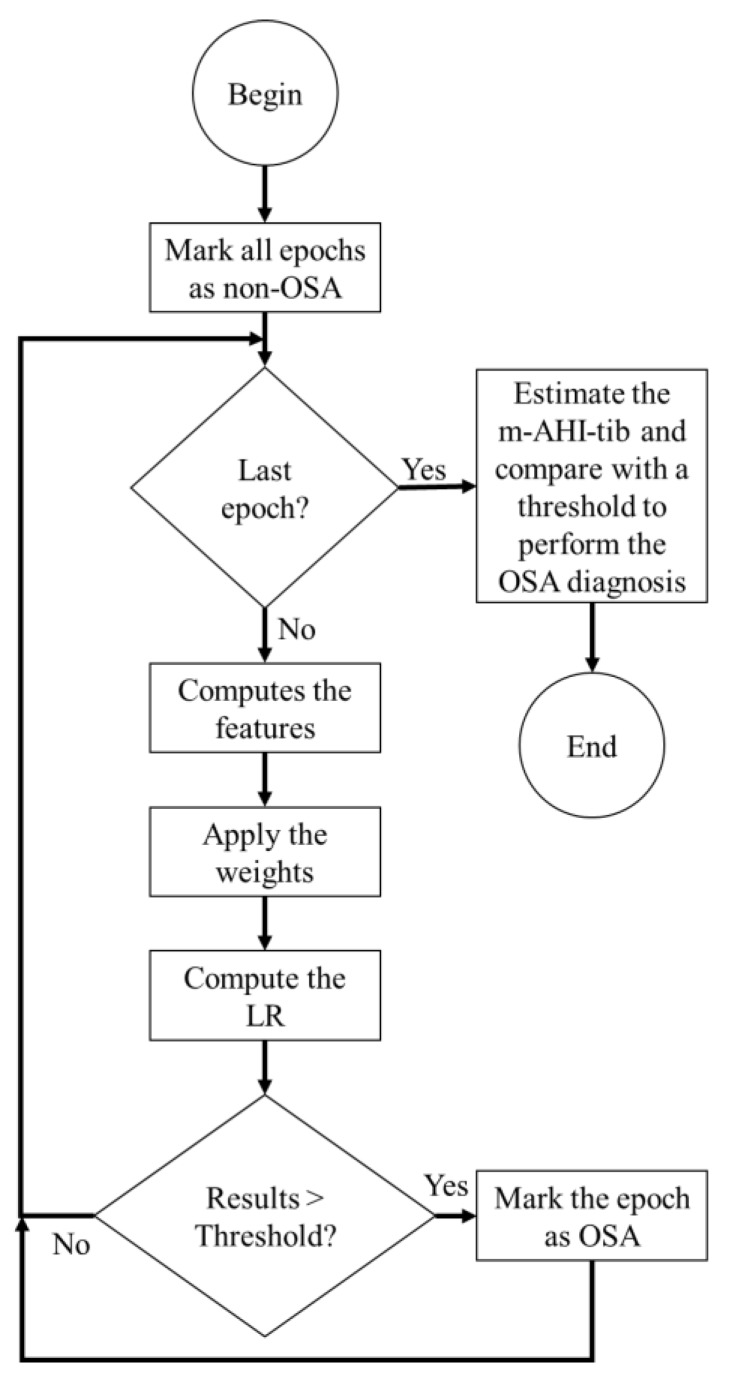
Flow diagram of the algorithm for the minute-by-minute OSA detection and for the global OSA diagnosis.

The process of feature creation, minute-by-minute classification and global diagnosis is presented in Figure 2. Five min (with a one min sliding) windows were used, therefore, the first and last two min of the data were always discarded. A terminal message was introduced at the end as last epoch, composed of only zeros with no physiological information. Two possible scenarios were considered:

(1) At the end of the test, if there are less than five min of data then this information was discarded and the terminal message was fed to the model;
(2) If at least five min of data were recorded then the classification begins at the third min and continues until the antepenultimate minute. The first and last two min were discarded, and the terminal message was fed to the model.

### 2.5. Performance Metrics

The performance of the minute-by-minute estimation was assessed by considering the average accuracy (Acc), sensitivity (Sen), specificity (Spe) and area under the receiver operating characteristic curve (AUC). The average global accuracy (Acc-G) was employed to evaluate the performance of the global OSA diagnosis.

## 3. Results

A feature selection process was employed to select the most relevant features for OSA detection. The process computed 50 iterations and each created an optimal feature set. Each optimal feature set was produced by sequential forward selection (SFS), an iterative process that considered the minimum average misclassification error (of the validation set) as the decision metric for the feature selection [19]. Specifically, the model starts by considering two sets, one empty (named optimal feature set) and one with all the features (named non-optimal feature set). In the first cycle of the SFS process, each feature was individually tested to assess which is more relevant (best value of the decision metric) and, therefore, moved from the non-optimal feature set to the optimal feature set. In the second cycle, each feature of the non-optimal feature set was tested, one by one, considering a new set with the previously chosen feature (in the first cycle) and the current feature under test. The feature whose model achieved the best value of the decision metric was moved from the non-optimal feature set to the optimal feature set. This process was repeated until none of the features tested in a cycle improved the decision metric, indicating that the most relevant optimal feature set was created. Afterward, the feature’s relevance was assessed according to the number of times they were selected to compose an optimal feature set and ordered according to their relevance, creating the ordered optimal feature set.

A second process was computed in an incremental way on the ordered optimal feature set with the goal of estimating the average error for each added feature. The final number of features was chosen by the minimum average error of the process that was repeated 50 times.

**Figure 3:**
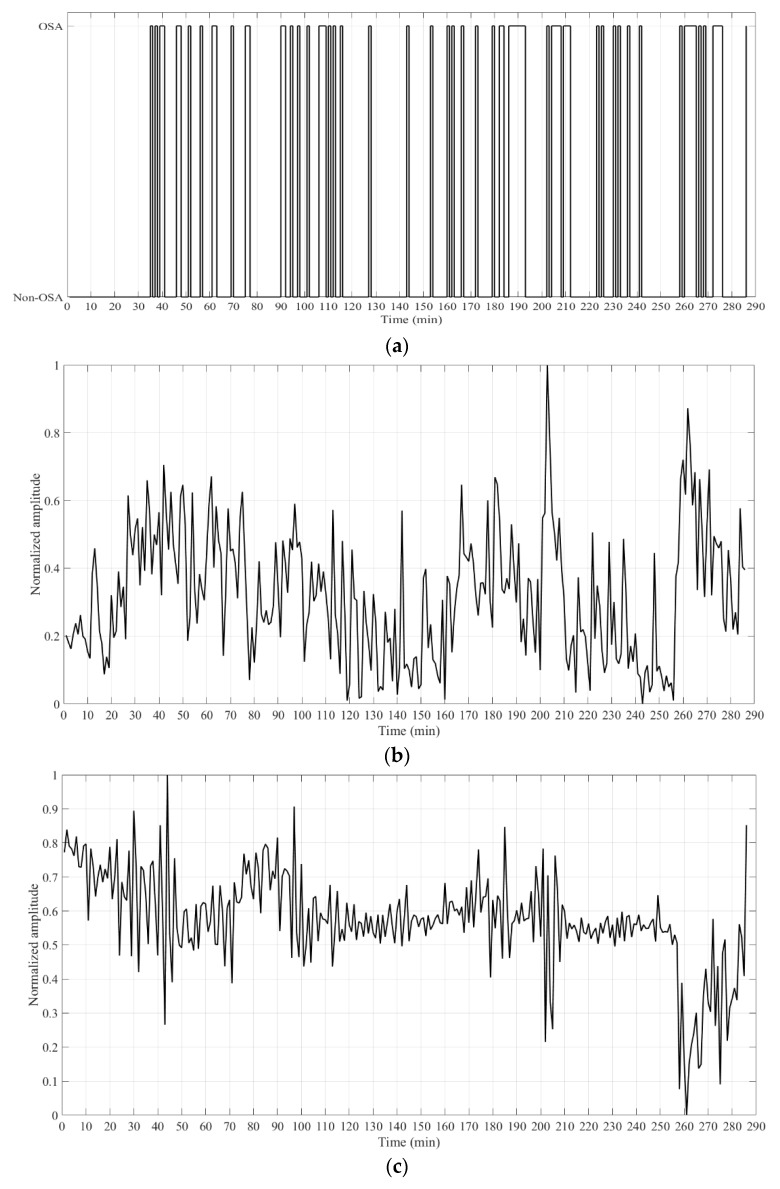

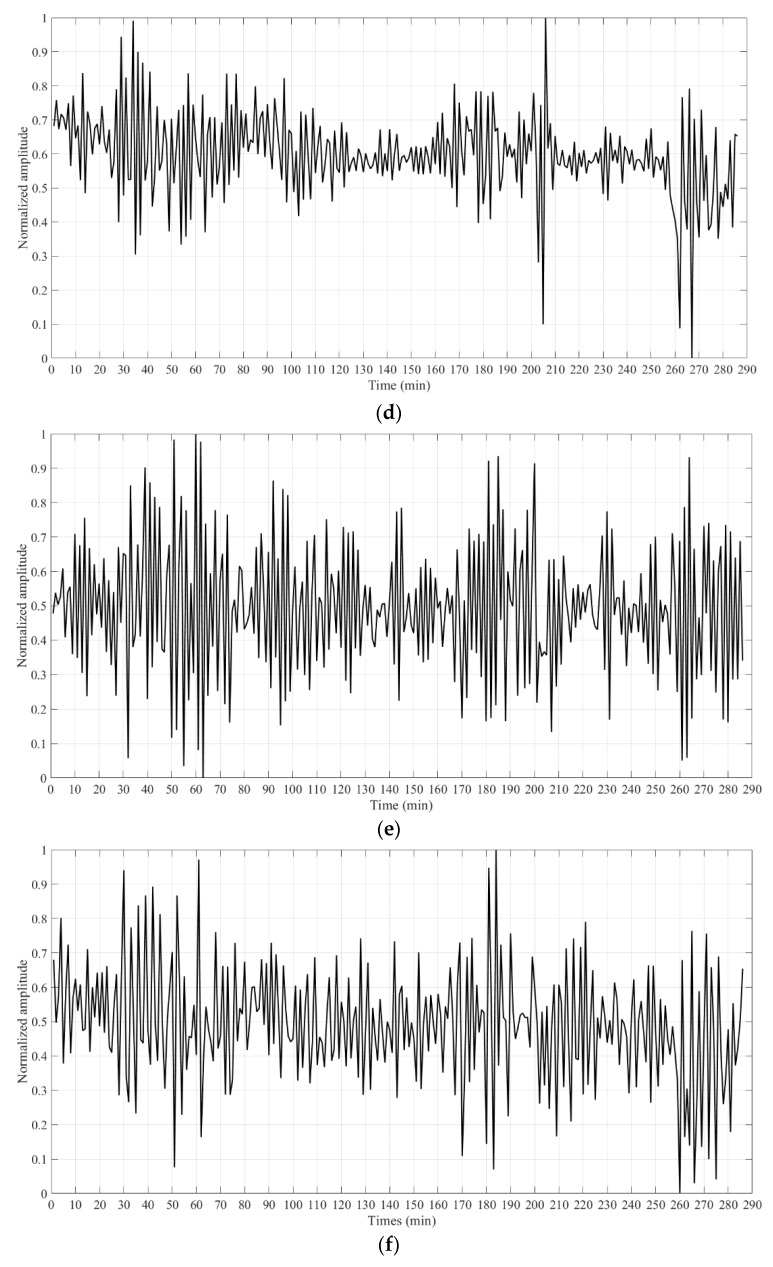
Example of the features selected by the sequential forward feature selection algorithm. (**a**) OSA label; (**b**) Variance of the central minute; Energy of the filters: (**c**) 2; (**d**) 3; (**e**) 8; (**f**) 9.

It was verified that the most relevant features were the variance of the central minute and the energy of the filters 2, 3, 8, and 9. An example of these features for one of the recordings is presented in Figure 3. It is possible to observe the variation of the feature when an OSA event occurred.

The performance of the algorithm is presented in Table 1. The results for the OSA detection are in the range of the methods reported in the state of the art that performed the analysis based on the SpO2 signal, where the Acc, Sen and Spe range, respectively, from 70% to 98%, 60% to 97%, and 69% to 100% [14]. The attained accuracy for the OSA diagnose is also in the range of the methods reported in the state of the art were the Acc-G range from 86% to 97% [14].

**Figure 4:**
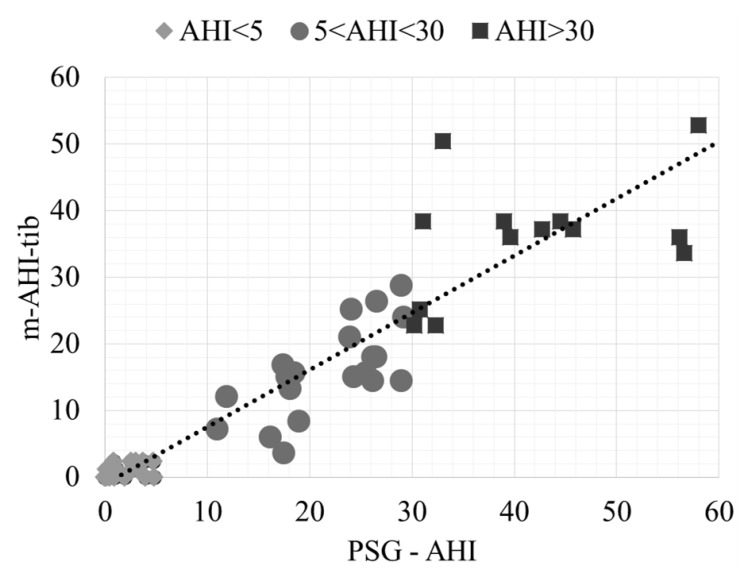
Regression plot of the AHI obtained by PSG and the predicted m-AHI-tib.

The regression plot of the AHI obtained by PSG and the predicted m-AHI-tib (AHI defined as the number of minutes with events per hour of time in bed [16]), for the employed dataset, is presented in Figure 4. The regression R^2^ was 0.87, supporting the validity of the technique for OSA diagnosis.

## 4. Discussion

Several approaches were proposed for OSA detection based on numerous source signals [14]. However, only the works based on the SpO2 signal analysis have interest for the comparison with the results achieved in this work. This evaluation is presented in Table 2.

A threshold-based method was proposed by Jung et al. [31] and it consists of the detection of three points. The first point marks a decrease in the SpO2 signal of greater than or equal to 1% and the second point is considered when the signal keeps decreasing to, at least, 3% lower than the first point. The final point happens when the signal grows back to either 3% above the second point or 1% below the first point. The total time between the first and third points must be between ten and 90 s. A Feedforward Neural Network (FFNN) was employed by Álvarez et al. [29] for OSA detection. The network was fed with features of the SpO2 signal, specifically: kurtosis, skewness, mean, relative power, spectral entropy, sample entropy, Lempel–Ziv complexity (LZC), and Central Tendency Measure (CTM). The same classifier was used by Almazaydeh et al. [23], fed by three inputs: oxygen desaturation index, delta index, and CTM. Mostafa et al. [25] also used a FFNN that was fed by seven features (from time and frequency domains) selected by a genetic algorithm from a set of 61 features. Marcos et al. [26] also employed a FFNN fed by CTM, LZC, and approximate entropy. The analysis of spectral features, attained on the 0.01 to 0.033 Hz band, was performed by Marcos et al. [28], using linear discriminant analysis for the classification. The same frequency band was also studied by Álvarez et al. [27] to create features and a genetic algorithm was employed to select the optimal features (from a set of features that also included LZC, sample entropy and CTM) to fed a LR. A wavelet decomposition method, implemented using the Haar wavelet, was presented by Morales et al. [30] using a k-nearest neighbor to perform the classification.

A deep learning approach was proposed by Mostafa et al. [22], feeding the raw SpO2 signal to a deep belief network to perform the OSA classification. A similar approach was employed by Pathinarupothi et al. [24], using a long short-term memory (LSTM) for classification.

By analyzing Table 2 it is possible to verify that Mostafa et al. [22] achieved the lower Acc when using the St. Vincent’s University Hospital/University College Dublin Sleep Apnea Database (UCD) dataset (with 25 subjects) but the highest Acc when using the apnea-ECG Database [32,33]. This could possibly indicate that the Apnea-ECG database does not have enough diversity of OSA events, due to the low number of subjects available with the SpO2 signal (8 subjects), thus leading to a high performance of the classifiers. A higher classification performance was also reported by Almazaydeh et al. [23], Mostafa et al. [25] and Pathinarupothi et al. [24] that employed the UCD dataset and the same conclusion can possibly be applied.

It is also possible to verify that the approaches based on deep learning attained the best results despite the small number of subjects of the database. However, these approaches are excessively complex to be efficiently implemented in the processing unit employed on this work, which has low computational resources, to make a minute-by-minute analysis while the LR-based classification requires far less computational resources (the order of magnitude of the number of parameters required for feature creation and classification is 10^2^).

A better performance, regarding the global classification, was achieved by Jung et al. [31] and Morales et al. [30]. However, the developed algorithm uses a simple classifier (LR) and easy to implement features. Both are relevant characteristics for a small and non-invasive HMD [13]. The other analyzed methods reported a lower global accuracy.

## 5. Development of the HMD

**Figure 5:**
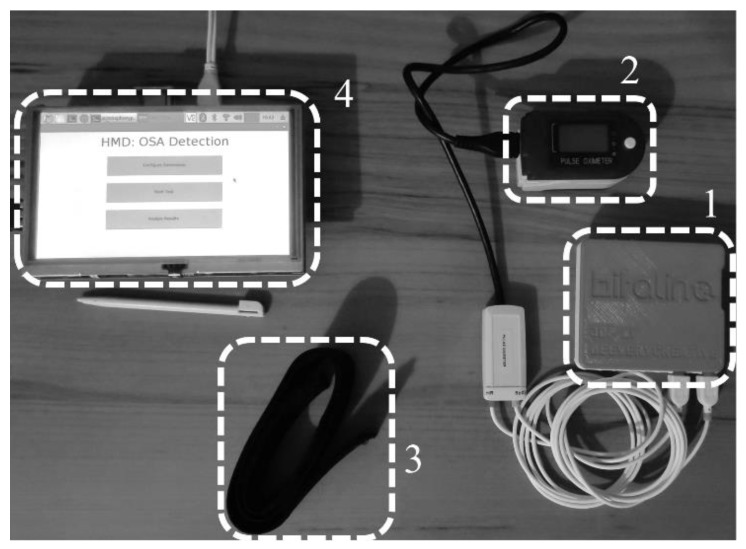
Hardware of the HMD. 1. Sensing unit, 2. Pulse oximeter, 3. Armband, 4. Processing unit.

A non-invasive HMD, whose architecture is presented in Figure 1, was developed to perform the OSA analysis. The device is composed of two units that communicate via Bluetooth and the employed hardware is presented in Figure 5.

The sensing unit was developed to be easily self-assembled. It was implemented by using the BITalino Core BT [34] that is composed of a microcontroller (ATmega328P), a communication module (Bluetooth communication) and a power management module that was fed by a 3.7 V lithium ion battery. The unit’s average load current is 50 mAh at it can last, at least, 17 h in real time acquisition over Bluetooth [34]. The sensing rate can be specified by the user in the processing unit (the device supports either 1, 10, 100 or 1 kHz; however, the default value of 100 Hz was employed since the measurements had fewer noise related artifacts than the measurements at 1 kHz) and the resolution of the signal (6 or 10 bit), is dependent upon the analog to digital conversion (ADC) port. For this work, only the 10 bit ports were used.

**Figure 6:**
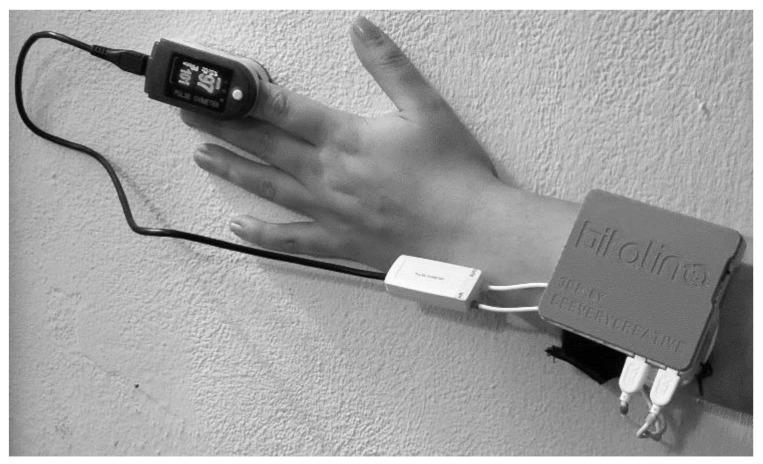
Sensing unit’s assembly.

The CMS-50D Plus pulse oximeter (Cooper Medical Supplies) was used to measure the SpO2 signal. The device can measure both SpO2 and heart rate signals. However, for this work only SpO2 was used. A representation of the sensing unit and pulse oximeter assembly is presented in Figure 6.

**Figure 7:**
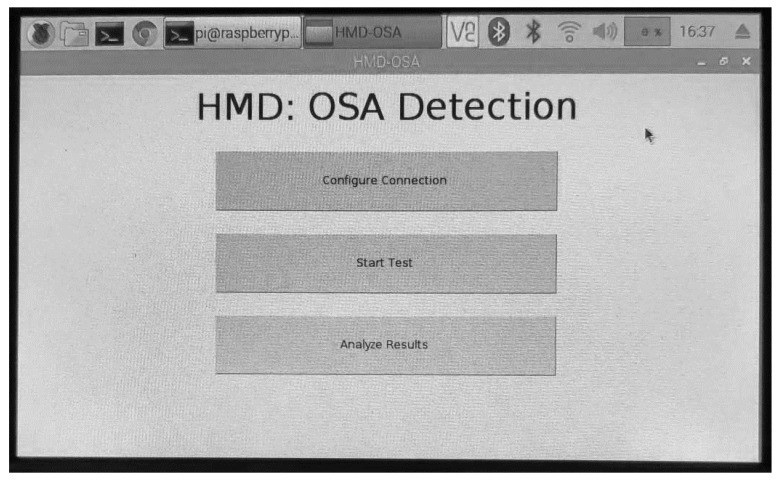
GUI of the processing unit’s application.

The processing unit is composed of a touch screen that displays the graphical user interface (GUI), presented in Figure 7, and a Raspberry Pi 3 B+ with a 64-bit ARM quad-core processor (1.4 GHz) that was fed by the direct current power supply. The two units automatically connect once the GUI is opened with the default bit rate (19,200 bit/s).

For the typical examination, the user is not required to change the default configurations and the procedure can be summarized in the following steps: (1) place the index finger in the pulse oximeter; (2) fasten the armband around the arm; (3) attach the sensing unit to the armband (through the s-shaped saddle clip that is behind the sensing unit); (4) connect the sensing unit; (5) connect the processing unit and wait until the GUI is open; (6) click on “Start Test” and a new window will pop-up with the “Stop Test” button (the sensing unit will begin the data transmission to the processing unit which in turn, will store the information in a text file with a timestamp); (7) click on “Stop Test” to finish the data collection (the communication between the units is finished); (8) click on “Analyze Results” and the application is fed the stored information to the developed OSA analysis algorithm (the results are stored in a text file); (9) the user can either examine the text files to check the results of the test or deliver the HMD to an expert that can verify the files. 

The processing unit cost was 60 € while the sensing unit cost was 240 € (165 € for the pulse oximeter and 75 € for the BITalino Core). A large variety of commercial SpO2 sensors is available on the market. However, all the validated SpO2 sensors, found in a market survey performed by the authors, have a price higher than 200 €. The price of the used sensor is lower than other validated commercial solution, thus, corroborating the choice of this work for a low cost HMD.

## 6. Conclusions

The main goal of this work was to develop an HMD that can perform the OSA diagnosis using only one sensor that is simple to self-assemble and non-invasive, allowing the test to be performed at the patient’s home. 

It was verified that the performance of the OSA assessment algorithm (epoch-based and global evaluation) is in the same range as the works available in the state of the art despite the less complexity of the proposed method. It was also verified that the m-AHI-tib has a strong correlation with the AHI measured by PSG, supporting the results attained in this work. Thus, the proposed model could possibly be employed for medical analysis, with the potential of increasing the accessibility of the population to the OSA diagnosis. 

The subsequent steps of this investigation are: an examination of other classifiers to assess if an improvement in the performance can be attained; validation of the HMD against a PSG to evaluate the performance of the implementation.

## Figures and Tables

**Table 1:** Average performance of OSA detection algorithm.

Acc-G (%)	Acc (%)	Sen (%)	Spe (%)	AUC
95	88	80	91	0.86

**Table 2:** Comparison of the attained results with the reported results from works available in the state of the art.

Work	Population (Number of Subjects)	Performance Metrics (Rounded Average Value)			
		Acc-G (%)	Acc (%)	Sen (%)	Spe (%)
[22]	25	-	85	60	92
[23]	8	-	93	88	100
[24]	8	-	96	-	-
[25]	8	-	98	99	97
[22]	8	-	98	79	96
[26]	83	86	-	-	-
[27]	144	87	-	-	-
[28]	113	88	-	-	-
[29]	127	90	-	-	-
[30]	79	94	-	-	-
[31]	92	97	91	83	89
This work	70	95	88	80	91
